# An Evaluation of the Clinical Evidence on the Role of Inflammation and Oxidative Stress in Smoking-Mediated Cardiovascular Disease

**DOI:** 10.4137/bmi.s480

**Published:** 2008-03-01

**Authors:** Adam Csordas, Georg Wick, Günther Laufer, David Bernhard

**Affiliations:** 1 Division of Experimental Pathophysiology and Immunology, Biocenter, Innsbruck Medical University, Fritz-Pregl-Str. 3, A-6020 Innsbruck, Austria; 2 Cardiac Surgery Research Laboratory of Autoimmunity, Department of Cardiac Surgery, Innsbruck Medical University, Innrain 66, A-6020 Innsbruck, Austria

**Keywords:** atherosclerosis, CRP, clinical study, ROS, heavy metals

## Abstract

The number of fatalities due to cardiovascular disease (CVD) continues to be far ahead of loss of human life caused by any other type of disease worldwide. According to the WHO, the annual global tobacco death toll is already 8.4 million and will reach 10 million by the year 2025. However, in contrast to other modifiable primary risk factors for CVD such as obesity, primary prevention strategies for smokers unable to quit are not available to date. This Review, by adopting the principles of evidence-based medicine, summarizes the most recent clinical studies on CVD in smokers, and concludes by suggesting a novel primary prevention strategy for CVD in smokers unable to quit. Evidence gathered from mechanistic studies involving basic research as well as large population-based approaches point to oxidative stress as the major insult imposed by cigarette smoke (CS), and a state of systemic inflammation, as signified by increased hs (high sensitivity) CRP levels in smokers, as the decisive pro-atherogenic response of the body to the initial insult. Since we identified oxidative stress induced by heavy metals as a significant pro-atherogenic activity of CS, strategies aimed at detoxifying heavy metals and combating inflammation appear as plausible approaches to counteract the accelerated onset of CVD in smokers. For this purpose, we discuss metal chelating agents and statins as promising novel primary prevention strategies in smokers unable to quit.

## Introduction

Smoking remains a major cause of cardiovascular disease (CVD)-related morbidity and mortality. The prediction of the WHO that by 2020 smoking will be the largest single health problem worldwide indicates the magnitude of burden that can be expected to be imposed on the health care system by smoking-associated morbidities. Considerable governmental efforts aimed at deterring people from cigarette smoke (CS) consumption have been put into action, and indeed western societies are now experiencing for the first time a situation in which the prevalence of smoking is on the decline. Despite this fortunate development, however, the percentage of young people who smoke remains at a high level, and the prevalence of smokers worldwide is still rising rapidly. Our finding that smoking constitutes the most significant risk factor for the development of early atherosclerosis in young people ( Knoflach and others, 2003 ) highlights the urgent need to reinforce preventive strategies aimed at reducing CS consumption. At the same time, robust biomarkers for identification of individuals at high risk for CS-associated atherosclerosis and associated cardiovascular events are urgently needed for timely instrumentation of primary prevention strategies, given that a vast majority of smokers are unable to quit their habit. Our current understanding of the mechanisms by which CS promotes atherogenesis allows targeting different types of molecules as possible candidates, the measurements of which can help determine whether there is an increased risk for cardiac events. At the same time, the quest for novel molecules that give an even more precise account of the presence of the disease and risk for atherosclerotic complications continues to proceed at a high pace. However, for a molecule to be considered a risk predictor, it needs to possess certain characteristics, especially if it is to be used for routine assessment in the clinical arena ( Mosca, 2002 ; Pearson and others, 2003 ), the most important of which include reliable standardization of the assay, association with CVD endpoints of clinical relevance, independence from classical risk factors and the ability to augment our current prognostic capabilities beyond that achievable by use of traditional risk stratification methods. Another important issue that needs to be addressed when referring to a biomarker for a certain disease is whether it serves as a risk marker, i.e. a measure for the disease process without any associated causal role, or as a risk factor i.e. is causally involved in the disease process, as the latter, besides being relevant in determining risk, also provides some insights into the underlying pathophysiology and potentially paves the way for the development of novel therapeutic strategies.

Since inflammation has been identified as a consistent driving force for all stages of the atherogenic process ( Ross, 1999 ), a host of markers for the grade of ongoing inflammation have been postulated to possess a strong predictive ability for the presence of progressing atherosclerotic changes in the vascular wall. Most significantly, inflammatory markers might indicate ongoing inflammation at a sub-clinical stage of atherosclerosis, rather than a clinically overt phenotype, which allows for identification of a cohort of people that would benefit from therapeutic and lifestyle intervention strategies.

In addition to markers pointing to a pro-inflammatory status, there is a large body of evidence in support of biomarkers indicative of a perturbed redox balance in the process of atheroma formation. We and others provided evidence for a reactive oxygen species (ROS)-mediated pathophysiological pathway conducive to atherogenesis that might have a role to play, in particular, in CS-mediated CVD ( Bernhard and others, 2003 ; Bernhard and others, 2005 ). Most of these types of molecules arise from oxidative modification of various lipid moieties, giving rise to lipid derivatives such as oxysterols, isoprostanes and oxLDL. Besides measurement of lipid oxidation products, the presence of oxidants such as free radicals and heavy metals can be directly determined by electron spin resonance (ESR)-spectrometry. In fact, data gained in the course of research carried out by our group support our working hypothesis that oxidative stress, generated by an interplay of metals and ROS, has a significant role to play in the pro-atherogenic activity of cigarette smoke extract (CSE) *in vitro* ( Bernhard and others, 2003 ; Bernhard and others, 2005 ; Csordas and others, 2006 ). Moreover, our finding of increased heavy metal levels in the serum of young smokers enabled extrapolation of our *in vitro* results to the *in vivo* situation ( Bernhard and others, 2006 ).

This review (i) summarizes the most recent data on biomarkers for CVD with emphasis on risk assessment in chronic smokers, (ii) relates the origin of these markers to the underlying pathophysiological cascade of events elicited by CS, and (iii) discusses the data in relation to our own concept of CS-induced atherosclerosis. Finally, (iv) we make an attempt to translate this data into a novel primary prevention strategy for the large population of smokers unable to quit their habit.

## Cigarette Smoke Consumption and Inflammation

### hsCRP

Evidence gathered from research involving basic experimental designs as well as population-based observational studies led to the recognition that inflammatory processes are central to all stages of the atherosclerotic disease, with inflammation being decisive for initiation, progression and onset of clinically overt disease ( Libby and others, 2002 ). Among the various biomarkers that are available for measuring ongoing inflammation, plasma CRP represents the most extensively studied one, and has a long history in the clinical arena as an acute-phase reactant, the quantification of which enables detection of the presence of an infectious disease. These clinically applied CRP-assays, however, were only able to detect massive elevations of circulating CRP and were not sensitive enough to accurately measure CRP levels in apparently healthy individuals below the threshold level that suggest an ongoing infection. Development of highly sensitive assays, however, ultimately permitted to reliably assess these low abundant CRP levels (hsCRP), so that variations in the conventional reference ranges of CRP, previously considered as normal, are now receiving increasing recognition as the most promising candidate biomarkers of chronic low grade inflammation and associated risk of cardiac events in otherwise healthy people. Initial data suggestive of a potential role of CRP as an independent predictor of cardiac events came from the European Concerted Action on Thrombosis and Disabilities (ECAT) study ( Anonymous, 1993 ) that established elevated CRP levels as a predictor for future coronary events in people with stable and unstable angina ( Haverkate and others, 1997 ). Notably, this study found markedly elevated levels of CRP among the smoking participants compared to the non-smokers. Later, several clinical studies extended these results to cohorts without manifest CVD by demonstrating that also among subjects with no prior occurrence of ischemic heart disease, plasma CRP levels serve as a prognostic marker for future cardiovascular events. Ridker and colleagues analysed the predictive capabilities of CRP as related to other known risk factors among participants of the Physicians’ Health Study and provided evidence for an independent predictive role of circulating CRP for coronary events and peripheral arterial disease in initially healthy subjects ( Ridker and others, 1997 ; Ridker and others, 1998a ). In another cohort of subjects participating in this study, hsCRP levels proved to augment the predictive information provided by lipid screening alone ( Ridker and others, 1998b ). An investigation involving the Augsburg cohort of the Monitoring Trends and Determinants in Cardiovascular Disease (MONICA) study ( Bothig, 1989 ) evaluated the role of CRP in cardiovascular risk prediction in initially healthy men on the basis of an 8-year follow-up, and confirmed the predictive relevance of serum CRP levels for occurrence of coronary heart disease in subjects without prior cardiac disease ( Koenig and others, 1999 ). This study concluded that subjects in the highest quintile of CRP distribution have a 2.6-fold increased risk for future CVD-related outcomes. Also in this study, a strong unadjusted correlation of smoking with elevated CRP levels was detected. Using data from the Women’s Health Study, Ridker et al. found that baseline plasma hsCRP constitutes an independent risk factor that, in combination with plasma LDL, better identifies women at increased risk for first cardiovascular events than implementation of cholesterol screening alone ( Ridker and others, 2000 ; Ridker and others, 2002 ). These findings are of particular interest, given that almost 50% of cardiovascular events occur in individuals with below average plasma levels of total cholesterol ( Braunwald, 1997 ), who at present are missed by current criteria for instrumentation of pharmacological therapy and lifestyle interventions.

Of note, in a secondary prevention population, pravastatin therapy has been shown to lower median plasma hsCRP in a manner independent of plasma cholesterol levels. Furthermore, risk reduction for recurrent cardiac events associated with statin therapy was more pronounced in individuals exhibiting a pro-inflammatory state ( Ridker and others, 1998d ; Ridker and others, 1999 ). Another double-blind prospective trial on a primary prevention cohort found that under pravastatin treatment, median CRP levels decreased by 16.9% in an LDL-C independent manner ( Albert and others, 2001 ). Confirmation of this result was obtained in a primary prevention cohort by implementation of lovastatin therapy in the course of the Air Force/Texas Coronary Prevention Study (AFCAPS/Tex-CAPS), as well as by administration of cerivastatin to patients with primary hypercholesterolemia—both drug regimens resulted in a significant reduction of plasma CRP levels ( Ridker and others, 2001b ). Of note, statin therapy proved to be effective in reducing risk for first coronary events in subjects with elevated CRP levels in the absence of overt hyperlipaemia ( Ridker and others, 2001b ). Interestingly, while there was a clear dose-response effect of cerivastatin on LDL-C, such an effect was not observed with regard to CRP, with a low-dose cerivastatin regimen being associated with a prominent reduction of median CRP levels ( Ridker and others, 2001c ). This appears to be a substantial finding, as it demonstrates that the anti-inflammatory effects of statins operate independently of their lipid-lowering activity, and that the decline of CRP levels is not secondary to statin-mediated modulation of the lipid profile. At the same time, these data suggest that primary prevention strategies for CVD, based on statin treatment, designed as an anti-inflammatory treatment in people identified as exhibiting a pro-inflammatory state might be feasible also from an economic point of view, given that even moderate doses of statins proved to be a successful strategy in combating inflammation.

However, it has to be pointed out that the overall data on an incremental prognostic information of hsCRP beyond that of traditional risk factors in predicting first major cardiovascular events are not entirely consistent, and the value of CRP as a useful clinical tool for refinement of the current level of risk prediction has been questioned by other large observational studies that found either no or only a moderate increase in risk prediction by hsCRP after adjustment for traditional risk factors. For instance, a large cohort study of participants of the sixth examination cycle of the Framingham Offspring Study failed to find any predictive value of hsCRP for first major cardiovascular events ( Wang and others, 2006 ), and further studies based on earlier cohorts of the Framingham Heart Study and the Atherosclerosis Risk in Communities (ARIC) study found only a moderate incremental value in risk prediction of CRP after adjustment for traditional risk factors ( Wilson and others, 2005 ; Folsom and others, 2006 ). Blankenberg and Danesh provided evidence along this line from another observational study that suggest only a moderate relative risk for future cardiovascular events for those in highest third of CRP levels ( Danesh and others, 2004 ; Blankenberg and others, 2006 ). In a recent effort to assess the current evidence basis for recommendation of measurement of hsCRP levels for CVD risk assessment, the Centers for Disease Control and Prevention and the American Heart Association came to the conclusion that determination of hsCRP levels appears reasonable as an adjunct approach to refining of risk stratification in people deemed at intermediate risk by means of traditional risk factors. This report also included a recommendation of cut-off points of CRP levels to be used in the clinical arena, with values <1 mg/L indicating low risk and values >3 mg/L suggesting high risk ( Pearson and others, 2003 ).

Elevated hsCRP levels have been consistently observed in smokers in various cross-sectional ( Das, 1985 ) and case-control studies. For instance, Rohde and colleagues embarked on a cross-sectional study amongst subjects recruited by the Phycicians’ Health Study and found that plasma CRP levels are significantly associated with smoking in a stepwise manner with the number of cigarettes smoked per day correlating with CRP levels ( Rohde and others, 1999 ). Mendall and colleagues confirmed this finding in another cross-sectional study showing an independent positive correlation between smoking status and CRP levels ( Mendall and others, 1996 ). No direct association between the numbers of cigarettes smoked per day and CRP levels was found in a cross-sectional study conducted by Tracy and colleagues among an elderly cohort of the Cardiovascular Health Study, although there was a strong correlation between lifetime exposure to cigarette smoke and CRP ( Tracy and others, 1997 ). It is noteworthy that the association between CRP and pack-years of smoking remained significant even in individuals who had stopped smoking for 30 years or more. This finding suggests that the inflammatory cascade elicited by CS might persist even in the absence of the original insult. Kuller and colleagues conducted a case-control study using serum samples from participants of the Multiple Risk Factor Intervention Trial (MRFIT) ( Anonymous, 1982 ) and found a significant association between plasma CRP levels and subsequent cardiac mortality. Of note, this association proved to be considerably stronger when analysis was restricted to smokers ( Kuller and others, 1996 ). A Swedish cross-sectional study that investigated the relationship between CRP, sub-clinical atherosclerosis and various cardiovascular risk factors found CS consumption to be the variable that showed the strongest association with CRP levels ( Hulthe and others, 2001 ).

In summary, these results suggest that by mediating as yet unidentified insults, CS consumption causes a chronic state of inflammation that might be directly related to subsequent elevated risk for cardiovascular diseases. Future prospective population-based studies will bring clarity to the partially inconsistent findings on the predictive value of hsCRP for cardiovascular events in the absence of traditional lipid-related risk factors. The Justification for the Use of Statins in Primary Prevention: An Intervention Trial Evaluating Rosuvastatin (JUPITER), a large long-term double-blind placebo-controlled intervention study, is currently under way to reliably assess whether people considered healthy by conventional criteria, but showing signs of inflammation as assessed by plasma hsCRP levels, would benefit from statin therapy ( Mora and Ridker, 2006 ). Confirmation of reduction of risk for CVD-related events by lowering of plasma CRP in people lacking elevated LDL levels will provide rigorous proof of the notion that inflammation has a leading and causative role to play in promoting CVD, and will pave the way for development of improved risk stratification algorithms in the setting of primary prevention that more profoundly acknowledge the association of a pro-inflammatory state with an elevated risk for cardiac events.

## Linking Cigarette Smoke Consumption to Inflammation

The population-based studies described in the chapter above make a strong case for a pro-inflammatory state in smokers. However, the initial pathophysiological mechanism responsible for instigation of pro-inflammatory signalling events by CS constituents has not been characterized. In the course of research carried out by our group in the past years, we could show that metal-catalysed protein oxidation constitutes a central pathway of CS-mediated endothelial cell damage *in vitro* and corroborated this result by a cross-sectional study involving young smokers ( Bernhard and others, 2005 ; Bernhard and others, 2006 ; Bernhard and Wang, 2007 ). Extension of this finding came from a Finnish study that found body iron stores correlating with levels of cholesterol oxidation products ( Tuomainen and others, 2003 ) and risk for myocardial infarction ( Salonen and others, 1992 ). A chronic state of oxidative stress generated by repeated deposition of metals in the vascular wall ( bu-Hayyeh and others, 2001 ) might serve as a mechanistic link between the pro-oxidant and pro-inflammatory state observed in smokers ( Bernhard and Wang, 2007 ). The pro-inflammatory state would in turn further aggravate the pro-oxidant state and *vice versa* . In this light, the finding of elevated inflammatory markers among smokers even years after smoking cessation further suggests that continuous deposition of various heavy metals present in CS and their persistence in the endothelial wall might serve as the decisive pro-inflammatory stimulus ( Tracy and others, 1997 ). Another hypothesis to explain the systemic pro-inflammatory and pro-oxidative state in smokers suggests chronic bronchitis, a condition commonly observed in smokers, to be the decisive source of persistent pro-inflammatory signalling events. For instance, a Finnish study demonstrated ischemic heart disease morbidity to be associated with morbidity due to respiratory bronchitis by showing that the presence of symptoms of respiratory disease predicts risk for ischemic heart disease ( Koskela and others, 2005 ). Confirmation of this result came from another Finnish study that examined the predictive ability of the presence of symptoms of chronic bronchitis for risk of first coronary events. The authors found that symptoms of chronic bronchitis predict risk for coronary disease independently from other risk factors ( Jousilahti and others, 1996 ). In summary, it remains to be established whether chronic bronchitis as induced by oxidative damage of the bronchi and alveoli by metals and ROS present in CS ( Bernhard and others, 2005 ) or direct deposition of heavy metals within the vascular wall serves as the decisive pro-inflammatory stimulus in smokers.

## Cigarette Smoke Consumption and Oxidative Stress: Role of Lipid and Protein Oxidation Products in Smoking-Mediated Cardiovascular Disease

### Oxysterols

A lipid parameter that has been suggested as an index for oxidative stress is the group of cholesterol oxidation products. Oxysterols are increasingly being recognized as potential causative agents in the development of atherosclerosis and have been shown to exert potent and diverse pro-inflammatory and pro-fibrotic effects on the vascular wall ( Leonarduzzi and others, 2002 ; Leonarduzzi and others, 2005 ). However, the overall literature on the issue as to whether increased plasma levels of oxysterols are associated with an increased risk for atherosclerosis contains equivocal reports. For instance, among more than 30 parameters assessed, Salonen and colleagues identified serum levels of 7β-hydroxycholesterol as the strongest predictor of a 3-year increase in carotid wall thickness ( Salonen and others, 1997 ), while another study failed to find any association between 7-ketocholesterol and peripheral vascular disease ( Dyer and others, 1997 ). On the other hand, indirect evidence in favour of a role of 7β-hydroxycholesterol in promoting atherogenesis comes from a study carried out by Ziedén and colleagues investigating the mechanism underlying the epidemiological observation that Lithuanian men have a 4-fold higher risk for CVD than their Swedish counterparts despite the presence of a similar pattern of traditional risk factors in both populations, as they found higher levels of 7β-hydroxycholesterol in the Lithuanian population ( Zieden and others, 1999 ). Further support for the view that oxysterols are related to atherosclerosis comes from data provided by Yasunobu and colleagues showing a positive correlation between oxysterols and coronary artery stenosis ( Yasunobu and others, 2001 ) in patients undergoing coronary angiography. Of note, this study found no correlation between smoking status and circulating levels of oxysterols. Elevated serum levels of oxysterols have, however, been observed in smokers ( Mol and others, 1997 ) in the course of another study.

In summary, it seems that the issue of whether smoking is associated with increased oxysterol levels and whether elevated levels of oxysterols contribute to adverse clinical outcomes cannot be reliably judged on the basis of the data available so far, and remains to be addressed in future population-based studies.

### F2-isoprostanes

The most recent index of ongoing lipid peroxidation was provided by the identification of F2-prostaglandin isomers (F2-isoprostanes) as free radical-mediated oxidative degradation products of arachidonic acid ( Morrow and others, 1990 ; Roberts and Morrow, 1994 ; Lawson and others, 1999 ) produced independently of cyclooxygenases. Of note, endogenous production of isoprostanes can be reliably assessed non-invasively by measuring their excretion in urine ( Awad and others, 1993 ). As for oxysterols, a variety of pro-atherogenic activities of F2-isoprostanes have been described including vasoconstriction and activation of platelets and monocytes (for review see ( Patrono and FitzGerald, 1997 )), which suggests that, besides reflecting the degree of oxidation, these types of compounds might also have the ability to translate oxidative stress into atherogenic processes in the vascular wall. Increased urinary excretion of 8-iso-PGF _2α_ has been shown in the context of various cardiovascular disease states that have been associated with oxidative damage such as coronary occlusion/reperfusion injury ( Delanty and others, 1997 ), diabetes mellitus ( Davi and others, 1999 ) and cigarette smoking ( Morrow and others, 1995 ; Reilly and others, 1996 ). Comparing the urinary excretion rate and serum levels of free and esterified F2-isoprostanes in 10 smokers with those of 10 non-smokers, Morrow and colleagues found that urinary excretion rate as well as serum levels of F2-isoprostanes were significantly higher in smokers as opposed to their non-smoking counterparts. Of note, both the free and esterified serum prostanoids dropped to significantly lower levels upon 2 weeks of smoking cessation ( Morrow and others, 1995 ). Confirmation and extension of these results comes from a study conducted by Reilly and colleagues in which the authors provide evidence for a significantly higher urinary excretion of F2-isoprostanes in smokers compared to non-smokers ( Reilly and others, 1996 ). In agreement with the study of Morrow, abstinence from smoking resulted in a significant decrease in urinary prostanoid excretion although the levels did not reach those of the non-smoking controls. Moreover, administration of vitamin C alone or in combination with vitamin E, but not vitamin E alone, proved to be an efficient strategy to combat the increased isoprostane levels, as a drop in urinary isoprostane secretion by an average of 29% was achieved. Similarly, a more recent study found significantly elevated isoprostane levels in serum, plasma and urine of smokers, and a rapid drop of the oxidation products in all of these three compartments upon cessation of smoking ( Pilz and others, 2000 ). These studies underscore the validity of the notion that an imbalance of oxidants and anti-oxidants is operative in smokers. However, the role of isoprostanes in cardiovascular risk prediction in relation to conventional risk factors has not been investigated as yet and awaits clarification in future clinical studies.

### Cigarette smoke consumption and formation of oxLDL molecules

Elevated levels of circulating oxLDL molecules have been shown to correlate with various clinical manifestations of atherogenesis including manifest CVD, though prognostic studies assessing the predictive abilities of oxLDL for a defined clinical endpoint are sparse and contradictory. For instance, Wallenfeldt and colleagues examined the predictive ability of oxLDL for progression of intima-media-thickness (IMT) and carotid plaque formation in a sample of 326 initially healthy subjects over 3 years of follow-up ( Wallenfeldt and others, 2004 ). The authors came to conclusion that plasma levels of oxLDL at baseline significantly predict both of these clinical parameters even when adjusted for conventional risk factors.

In a prospective study within the setting of secondary prevention, Shimada and colleagues also showed that levels of oxLDL independently predict future cardiac events in patients with documented coronary artery disease ( Shimada and others, 2004 ). In agreement with these results, Meisinger and colleagues reported on a nested case-control study among participants in the MONICA project, in the course of which the authors identified oxLDL as a significant predictor for future coronary events in initially healthy people ( Meisinger and others, 2005 ). However, the largest prospective study on the prognostic value of oxLDL for coronary artery disease in apparently healthy people seriously questioned these results on the basis of the finding that the calculated relative risk associated with elevated oxLDL levels loses statistical significance after adjustment for standard risk factors. The authors found that oxLDL failed to give prognostic information beyond that provided by apolipoprotein B and the total cholesterol/HDL-C ratio, whereas the latter parameters remained significant predictors for risk for cardiac events when adjusted for oxLDL ( Wu and others, 2006 ). This result evidently challenges an independent contributory action of oxLDL to CVD development, and suggests that oxLDL rather serves as a surrogate parameter for apolipoprotein B and total LDL levels.

Circulating oxLDL molecules have been observed to be elevated in smokers by means of cross-sectional studies ( Fickl and others, 1996 ; Liu and others, 2000 ). However, further studies are needed to establish whether measurement of circulating oxLDL levels indeed augments our current predictive capabilities beyond that achievable by consideration of conventional risk factors, and whether therapies tailored to combat oxLDL levels are justified.

### Cigarette smoke consumption and formation of protein oxidation products

In contrast to the plentiful literature examining the utility of lipid peroxidation products as quantitative indices for oxidative stress, oxidative protein modifications in human plasma have been only sparsely investigated as potential biomarkers of oxidative damage. Proteins are, however, firmly recognized as major targets of oxidative attack *in vivo* ( Stadtman and Oliver, 1991 ). This knowledge, coupled with the long half-life of some proteins that enables accumulation of a certain oxidation product over time, suggests that protein oxidation products have the potential to serve as reliable and sensitive markers for oxidative stress ( Dean and others, 1997 ). As reviewed in great detail by Davis and colleagues ( Davies and others, 1999 ), radicals can give rise to an array of protein oxidation products that differ in their individual utility as biomarkers for oxidative damage for various biochemical reasons. Some of these compounds such as 3,4-dihydroxyphenyalanine (DOPA) or dityrosine have been found at an increased level in advanced human atherosclerotic plaques ( Fu and others, 1998 ). However, a detailed understanding of the nature of the different protein oxidation products and appropriate methods for their detection have only been developed recently, and therefore studies examining the association of certain protein oxidation products with pathophysiological endpoints relevant for CVD are sparse. As against this, the quantification of protein carbonyl formation as a generic marker for oxidative modifications of protein side-chains has been used for quite a while and is widely recognized as a valid approach to assess the overall extent of protein oxidation ( Stadtman and others, 1992 ). Using this technique, we confirmed in a recent study that oxidative damage takes place in endothelial cells subjected to CS *in vitro* ( Schmieder and others, 2006 ). In an *in vivo* study, increased protein carbonyl formation was shown in the plasma of smokers ( Pignatelli and others, 2001 ).

Another marker of oxidative damage that has been associated with chronic inflammatory disorders is generated by nitration of tyrosine residues of proteins ( Kaur and Halliwell, 1994 ). 3-Nitrotyrosine is created in the presence of nitrating agents (such as peroxynitrite, a major radical present in CS) and may hence be an especially promising candidate marker to assess the extent of oxidative damage in smokers. Moreover, since the nitration reaction of tyrosine is irreversible, 3-nitrotyrosine levels might provide an integrative summary of cumulative protein damage acquired over a certain time frame. 3-Nitrotyrosine levels have been shown to be significantly elevated in plasma, in the apolipoprotein B part of isolated LDL molecules as well as in the vascular tissue of smokers ( Petruzzelli and others, 1997 ; Beckman and others, 2004 ; Yamaguchi and others, 2005 ). The hypothesis that peroxynitrite of CS is decisively involved in mediating oxidative damage has also been confirmed *in vitro* by the detection of 3-nitrotyrosine formation in LDL particles exposed to CSE ( Yamaguchi and others, 2002 ). Discussing the clinical consequences of elevated 3-nitrotyrosine levels, Shishehbor and colleagues embarked on a case-control study to evaluate its predictive power for the presence of CVD, and found, after adjustment for the Framingham criteria and plasma CRP levels, a significantly higher odds ratio for coronary heart disease in the upper quartiles of 3-nitrotyrosine levels compared to the lowest quartile ( Shishehbor and others, 2003 ).

Overall, it would appear that the pattern of protein carbonyl or 3-nitrotyrosine formation provides a reliable footprint of accumulated oxidative damage, and the evidence on protein oxidation with regard to CS consumption is in line with the concept of a pro-oxidant state of smokers as identified by measurement of lipid peroxidation products. Moreover, preliminary data suggest that oxidative protein damage by nitric oxide-derived radicals might be a contributing factor to cigarette smoke-induced atherogenesis ( Shishehbor and others, 2003 ).

## Cigarette Smoke Consumption and Soluble Adhesion Molecules

Recently, increasing information on the ability of soluble adhesion molecules to reflect and promote CVD from both basic and clinical studies has become available, and plasma levels of adhesion molecules have been suggested to serve as promising candidate markers for the risk for developing atherosclerosis and associated complications ( Mulvihill and others, 2002 ; Blankenberg and others, 2003 ). Several population-based studies indeed provided evidence for a predictive ability of certain types of adhesion molecules for severity of ongoing atherosclerosis and risk for cardiac events. The Atherosclerosis Risk in Communities (ARIC) study provided the first evidence basis in support of the concept that adhesion molecules serve as novel independent biomarkers for atherosclerosis by showing that sICAM but not sVCAM levels predict risk for incident coronary heart disease (CHD) after adjustment for traditional atherosclerosis risk factors ( Hwang and others, 1997 ). Notably, this study found significantly higher levels of sICAM in smokers and a strong correlation between sICAM and years of cigarette smoking. This finding extends to the *in vivo* situation the *in vitro* -based data previously delivered by Kalra and colleagues who found elevated ICAM-1 expression in HUVEC treated with CS condensate ( Kalra and others, 1991 ). Further clinical studies in support of a predictive value of sICAM with regard to CVD-related endpoints came from Ridker and colleagues who embarked on a nested case-control study using data from the Physicians’ Health Study and found sICAM to predict future myocardial infarction independently of classical risk factors ( Ridker and others, 1998c ). Of note, also this study found significantly higher sICAM levels in smokers as compared to non-smokers. A cross-sectional analysis of an array of markers of inflammation in participants of the Women’s Health Study conducted by Bermudez and colleagues found circulating levels of various inflammatory markers including hsCRP, IL-6, sICAM and P-selectin to be significantly elevated in smokers ( Bermudez and others, 2002 ). Notably, levels of all inflammatory markers increased in parallel with higher degrees of smoking exposure. Statistical significance of all markers but P-selectin was maintained after adjustment for conventional lipid- and non-lipid-associated CVD risk factors. Another case-control study involving participants from the Women’s Health Study found a significant association between elevated soluble P-selectin levels and CS consumption ( Ridker and others, 2001a ). In this study, the predictive ability of P-selectin for future cardiac events attained statistical significance even after adjustment for classical risk factors. This result extrapolates to the *in vivo* setting our own *in vitro* finding of a shedding of P-selectin into cell culture medium from HUVEC exposed to CS ( Bernhard and others, 2005 ). Further evidence in support of pro-adhesive events elicited in the vascular wall by CS constituents has been provided by Mazzone and colleagues who found sICAM and sVCAM to be significantly elevated in hypertensive smokers as compared to non-smoking hypertensive patients ( Mazzone and others, 2001 ), a finding that further underlines the independence of smoking from other known risk factors for CVD. Blann and colleagues performed a cross sectional study on inflammatory markers in regular smokers and found soluble von Willebrand factors (svWf), sICAM and sP-selectin to be significantly elevated amongst the smokers ( Blann and others, 1997 ). Remarkably, sICAM and sP-selectin dropped to significantly lower levels after a 6-week smoking cessation.

In summary, there is a consistent association between CS consumption and elevated sICAM levels. This finding is of particular interest as it highlights a boost of pathophysiological processes that are at play at the earliest stages of atherogenesis in people consuming CS. An up-scaling of inflammatory processes in the vascular wall by CS-derived compounds by their acting as a stress factor to endothelial cells appears to be a plausible mechanistic link between smoking and accelerated atherogenesis.

## Conclusion

**Figure 1:**
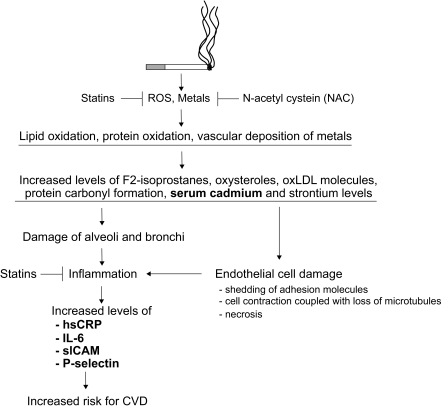
Smoking leads to oxidative damage of lipids and proteins with each drag of CS inhaled. At the same time, heavy metals enter the blood *via* the lungs and are deposited within the vascular wall. Metal-catalysed oxidation reactions lead to a systemic pro-inflammatory state that is associated with an increased risk for cardiovascular events. A measure of the inflammatory state can be obtained by assessing circulating hsCRP, IL-6, ICAM and P-selectin levels. Furthermore, markers that serve as a direct footprint of the extent of overall lipid and protein damage are amenable to CVD risk assessment. The pro-inflammatory cascade of events might be abrogated by either combating inflammation by means of metal chelating agents such as NAC, or by compounds that target the pro-inflammatory response to the insult imposed by CS such as statins. The latter group of drugs also has anti-oxidative properties, and thus might prove to be effective in particular against smoking-mediated CVD. All the markers depicted in the graph have been shown to be present at an increased level in the blood of smokers. The markers presented in bold have been shown to predict risk for future CVD independent of conventional risk factors.

CS consumption remains a significant factor in the development of atherosclerosis, the associated complications of which exact as large a toll on health and life of human populations as no other disease currently afflicting mankind. CS consumption has been correlated, using cross-sectional as well as prospective case-control studies, with elevated levels of biomarkers for oxidative stress such as isprostanes, oxysterols and oxLDL molecules, and inflammatory markers including hsCRP, IL-6 and soluble adhesion molecules. Our finding of increased levels of heavy metals in the serum of young smokers defines an additional biomarker that is elevated in smokers. These data support our working hypothesis of CS-induced atherosclerosis, namely that oxidative stress generated by Fenton-like catalytic reactions between ROS and the high content of heavy metals present in CS is operative in people exposed to CS ( Fig. 1 ). However, as has been described in the chapters above, although there is a large body of evidence pointing to elevated levels of various markers of oxidative stress in smokers, prospective studies on the predictive value of these markers for clinical cardiovascular endpoints are sparse and have yielded inconsistent results. At the same time, studies that have been undertaken so far to analyse the effects of antioxidant supplementation as a preventive measure in smokers concluded with partially equivocal results. Given that the blood concentration achievable by supplementation with the antioxidants administered has not been tested in most cases, and their long-term impact on endpoints of clinical relevance has not been evaluated, caution is needed in the interpretation of these results. Thus, it would be wrong to conclude that oxidative stress does not play a major role in smoking-mediated CVD. The approach of applying pure anti-oxidants neglects our finding of increased levels of heavy metals as a potential source of ROS in smokers as metals are not targeted by the vitamins studied. Interventional studies applying metal chelating agents that focus on clearly defined cardiovascular endpoints are urgently needed to make a final verdict on the value of these compounds as a preventive strategy for CVD in smokers. Potential candidate compounds appear to be sulphuric compounds such as N-acetylcysteine that has already been shown by us to partially inhibit CS-induced endothelial cell damage *in vitro* ( Bernhard and others, 2005 ). Another therapeutic option one might want to consider is administration of a low-dose statin regimen, given the finding that statins have the ability to combat oxidative stress and inflammation concomitantly even at moderate doses that do not influence the blood lipid pattern.

In summary, there is no doubt about the multi-factorial background of CS-induced atherogenesis, and the relative contributions of inflammation and oxidative stress to this phenomenon still remain to be clarified. However, the pro-atherogenic lines of events are not mutually exclusive, with inflammation causing oxidative stress and *vice versa* . Furthermore, preliminary evidence points to a protective action of compounds that combat both inflammation and oxidative stress such as statins. It would thus appear that as a primary prevention strategy against CVD, we have some promising tools at hand. Long-term interventional trials are warranted for examining in detail their efficacy.
